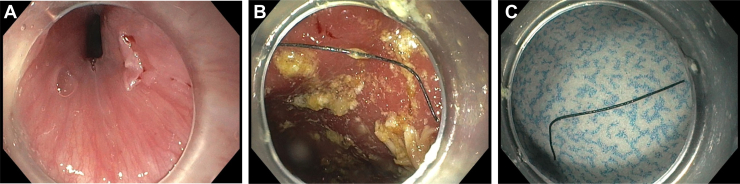# Dangers of an American Pastime: Grill Wire Bristle Ingestion Masquerading as a Bony Impaction

**DOI:** 10.1016/j.gastha.2025.100875

**Published:** 2025-12-30

**Authors:** Clive Jude Miranda, Casey Marie DeBeltz, Aun Raza Shah

**Affiliations:** Department of Gastroenterology, CHI Health Creighton University Medical Center – Bergan Mercy, Omaha, Nebraska

A 33-year-old healthy female presented 3 hours after eating a boneless pork chop made off her backyard propane grill with the sensation that she may have swallowed a bone. She was, however, adamant that the meat was boneless. She reported odynophagia and noted sharp discomfort in the superior chest just above the sternal angle of Louis. She had no vocal changes, abdominal pain, or difficulty handling secretions. An x-ray of the neck was negative for any obstruction or extraluminal air and a gastrointestinal cocktail provided minimal relief.

An upper endoscopy within the hour revealed a nonbleeding superficial mucosal tear at the level of the cricopharyngeus muscle (Figure A). The stomach contained a large amount of food residue. At the antrum, a thin, sharp foreign body 1.5 cm in length was found (Figure B). Cap-assisted removal was accomplished with Raptor forceps. Further inspection revealed this to be a metallic bristle of a wire grill brush (Figure C).

Outdoor grilling is a common component of the American pastime. It is not uncommon to utilize wire brushes to clean these devices; however, dislodged bristles have a propensity to adhere to grilled food and cause severe complications, including intestinal perforation, vascular injury, and death. Nearly 1700 Emergency Department visits in the United States between 2002 and 2014 were due to bristle consumption. Gastroenterologists suspect bony ingestion when patients present with dysphagia and sharp pain after having consumed meat products. However, it is also imperative to note how the food was cooked as even vegetarian items could have a wire bristle lodged in them during grilling and inadvertently consumed.

**Figure:**